# Supplementary material for: Sex-specific evolutionary programs shape recombination rate evolution in house mice
Source: Genetics. 2025 Nov 14;232(1):iyaf251. doi: 10.1093/genetics/iyaf251 (PMC12774844; doi:10.1093/genetics/iyaf251)
Supplement: iyaf251_Supplementary_Data [file iyaf251_supplementary_data.zip › Figure_S5_GENETICS-2025-308628.pdf]

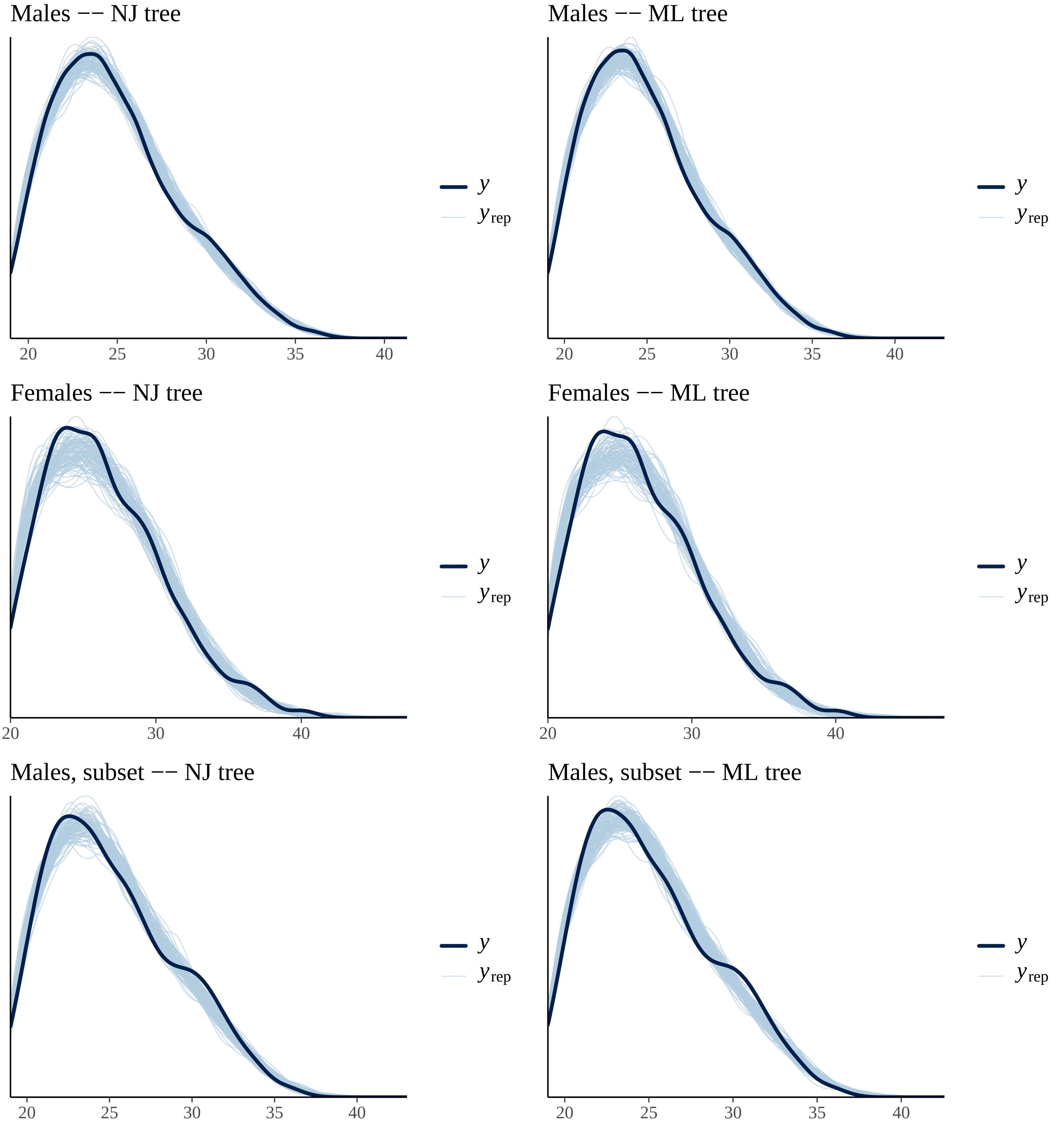
Males −− NJ tree
Males −− ML tree
Females −− NJ tree
Females −− ML tree
Males, subset −− NJ tree
Males, subset −− ML tree
y
y rep
20
25
30
35
40
