# Supplementary material for: Sex-specific evolutionary programs shape recombination rate evolution in house mice
Source: Genetics. 2025 Nov 14;232(1):iyaf251. doi: 10.1093/genetics/iyaf251 (PMC12774844; doi:10.1093/genetics/iyaf251)
Supplement: iyaf251_Supplementary_Data [file iyaf251_supplementary_data.zip › Supplemental_Figure_Legends_GENETICS-2025-308628.docx]

**SUPPLEMENTARY FIGURE LEGENDS**

**Figure S1.** Global distribution of strains included in this study. The map is color-coded according to approximate *Mus musculus* subspecies ranges. Four strains — SPRET/EiJ, PANCEVO/EiJ, Mus pahari/EiJ (PAHARI), and CAROLI/EiJ — derive from the contraspecific species *M. spretus*, *M. spiceligus*, *M. pahari*, and *M. caroli*, respectively. These species are sympatric with *M. musculus* and are indicated with red labels.

**Figure S2.** Comparison of MLH1 foci counts across independent studies. Boxplots display the number of autosomal MLH1 foci in spermatocytes from strains (A) CAST/EiJ, (B) outbred Gough Island mice, (C) LEWES/EiJ, (D) MOLF/EiJ, (E) PWD/PhJ, (F) SPRET/EiJ, and (G) WSB/EiJ. Individual data points are overlayed on the boxplots and jittered for ease of visualization. *P*-values were calculated from Mann-Whitney U-tests. Significant, but quantitatively modest, differences in MLH1 foci counts are observed for Gough, PWD/PhJ, and WSB/EiJ.

**Figure S3.** Density distribution of observed MLH1 foci counts in (A) males and (B) females superimposed against simulated normal and Poisson distributions truncated to exclude values <19 (males) and <20 (females). Simulated data were generated using sex-specific empirical estimates of the mean and variance in MLH1 foci counts.

**Figure S4.** Diagnostic plots for estimated model parameters. Plots are facetted by the MLH1 foci count dataset and the phylogeny used to account for strain relatedness in the fitted model. Within each subpanel, plots on the left display the estimated posterior distribution of the parameter, aggregated across MCMC chains. The smooth, unimodal distributions with consistent shapes across chains indicate stable sampling. Plots on the right show the sampled values across iterations for each MCMC chain. Each colored line represents a separate chain. The high level of visual overlap across chains indicates good mixing and convergence. The “Males, subset” plots correspond to the male MLH1 foci dataset that was down sampled to include only the subset of strains included in the female dataset.

**Figure S5.** Density plots for the observed distribution of MLH1 foci counts (black line) and 100 data sets simulated from the posterior distribution of the specified model (light blue lines). Plots were generated using the pp_check function in the R package brms. Plots are facetted by MLH1 foci count dataset and the phylogeny used to account for strain relatedness. The “Males, subset” plots correspond to the male MLH1 foci dataset that was down sampled to include only the subset of strains included in the female dataset.

**Figure S6.** Simulated posterior distribution of phylogenetic heritability, $H_{P}^{2}$. Plots are facetted by the MLH1 foci count dataset and the phylogeny used to account for strain relatedness. Parameter values were simulated from the posterior distribution using the as_draws_df function in the brms package for R. Simulated variance levels were then used to compute simulated $H_{P}^{2}$ values. The vertical red line corresponds to the median $H_{P}^{2}$ value. The “Males, subset” plots correspond to the male MLH1 foci dataset that was down sampled to include only the subset of strains included in the female dataset.

**Figure S7.** Neighbor joining phylogenetic tree for genetically diverse inbred mouse strains. Tree is rooted to the outgroup species *M. pahari* represented by inbred strain PAHARI/EiJ. For brevity, strain labels at the tips of the tree exclude laboratory codes. Wild-derived inbred strains from *M. musculus* subspecies are labeled and color-coded in gold (*domesticus*), red (*castaneus*), gray (*molossinus*), and turquoise (*musculus*). Strain SPRET is a wild-derived inbred strain of *M. spretus* origin, CAROLI is a representative of the *M. caroli* species, and PANCEVO is a wild-derived inbred strain of *M. spiceligus*. Panels to the right of the tree plot average MLH1 foci counts (±1 standard deviation) for males and females for each strain, as well as the difference between male and female MLH1 foci counts (±1 standard deviation). Note that MLH1 foci counts are not available for females from all strains. Figure made using the ggtree package for R with aesthetic modifications in BioRender (Dumont, B. (2025) https://BioRender.com/bsenvl5).
