# Supplementary figures and images for: Sex-specific evolutionary programs shape recombination rate evolution in house mice
Source: Genetics. 2025 Nov 14;232(1):iyaf251. doi: 10.1093/genetics/iyaf251 (PMC12774844; doi:10.1093/genetics/iyaf251)

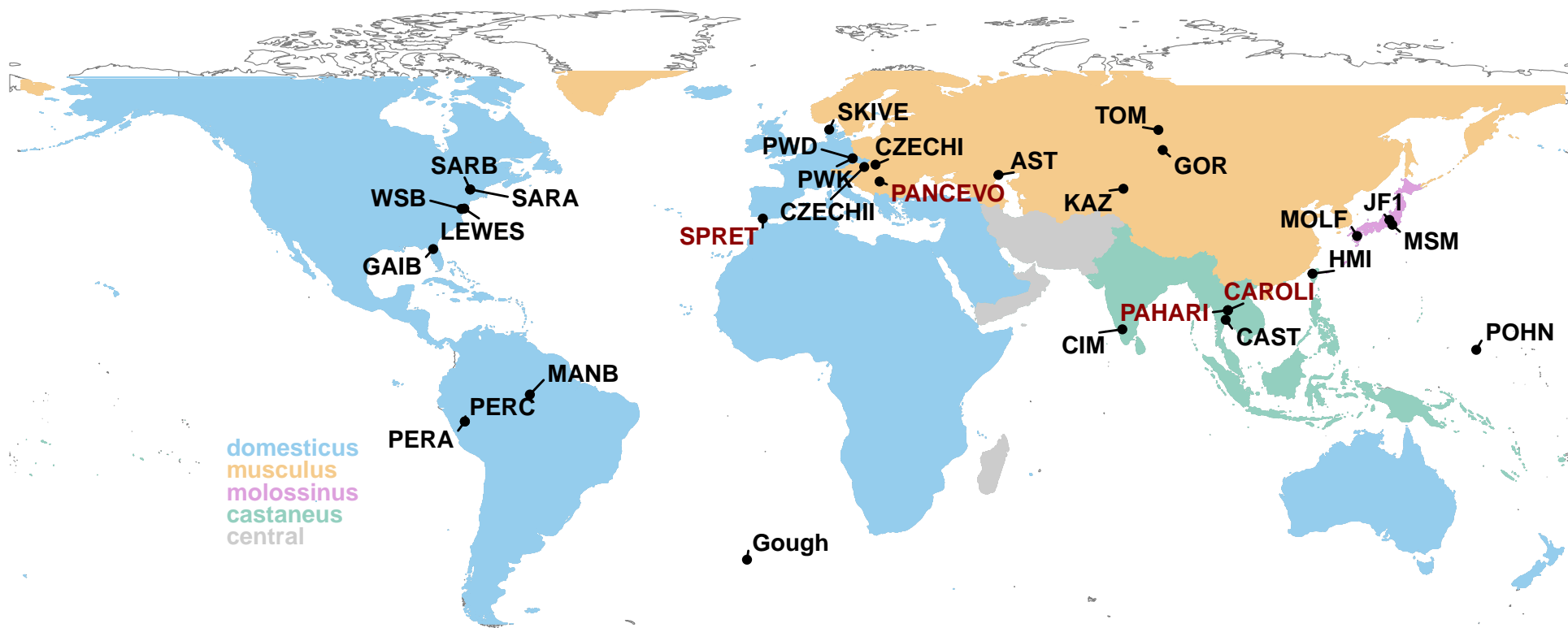

Supplement: iyaf251_Supplementary_Data [file iyaf251_supplementary_data.zip › Figure_S1_GENETICS-2025-308628.pdf]

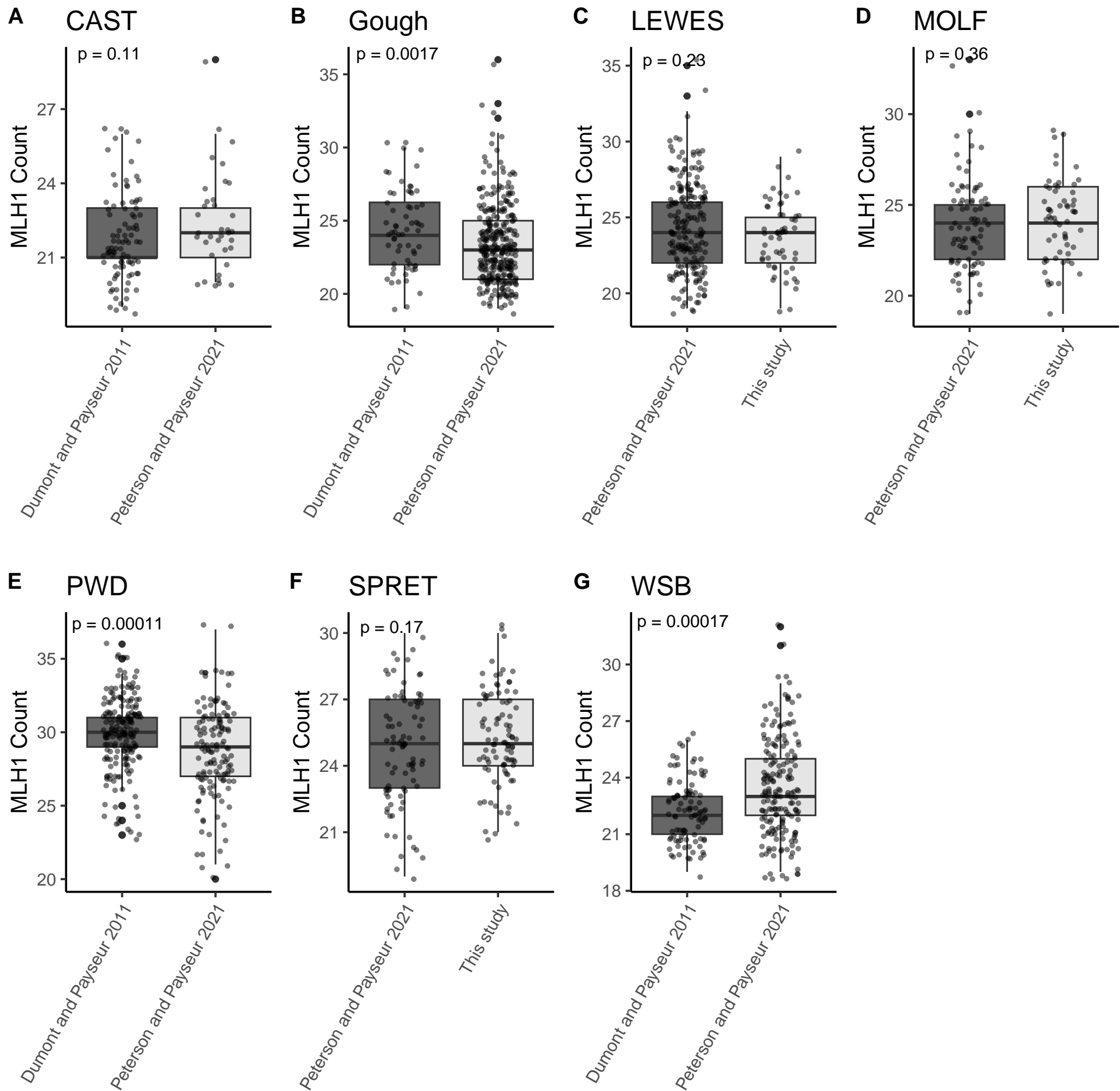

Supplement: iyaf251_Supplementary_Data [file iyaf251_supplementary_data.zip › Figure_S2_GENETICS-2025-308628.pdf]

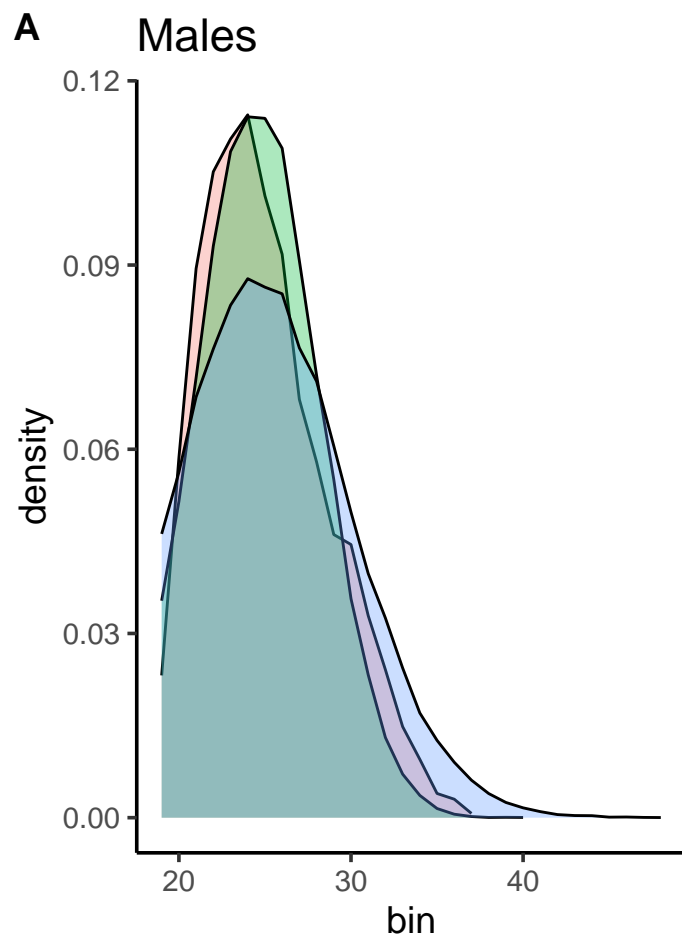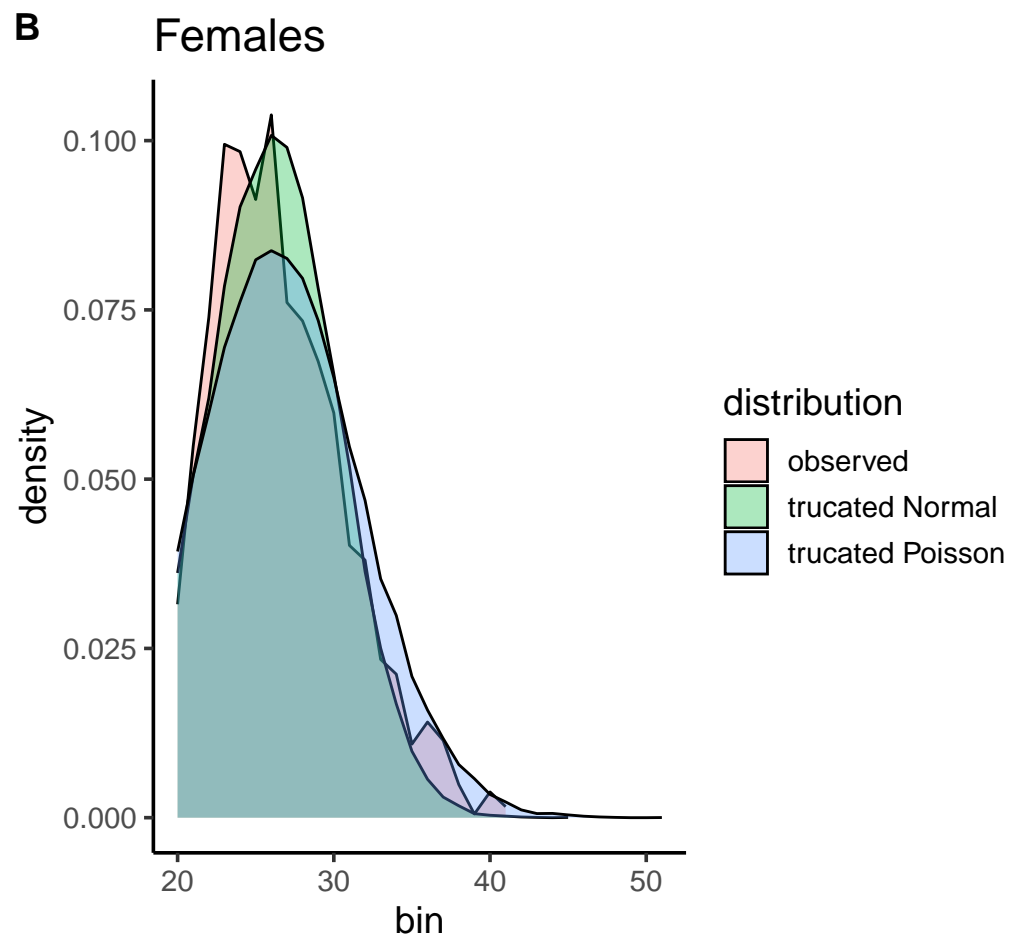

Supplement: iyaf251_Supplementary_Data [file iyaf251_supplementary_data.zip › Figure_S3_GENETICS-2025-308628.pdf]

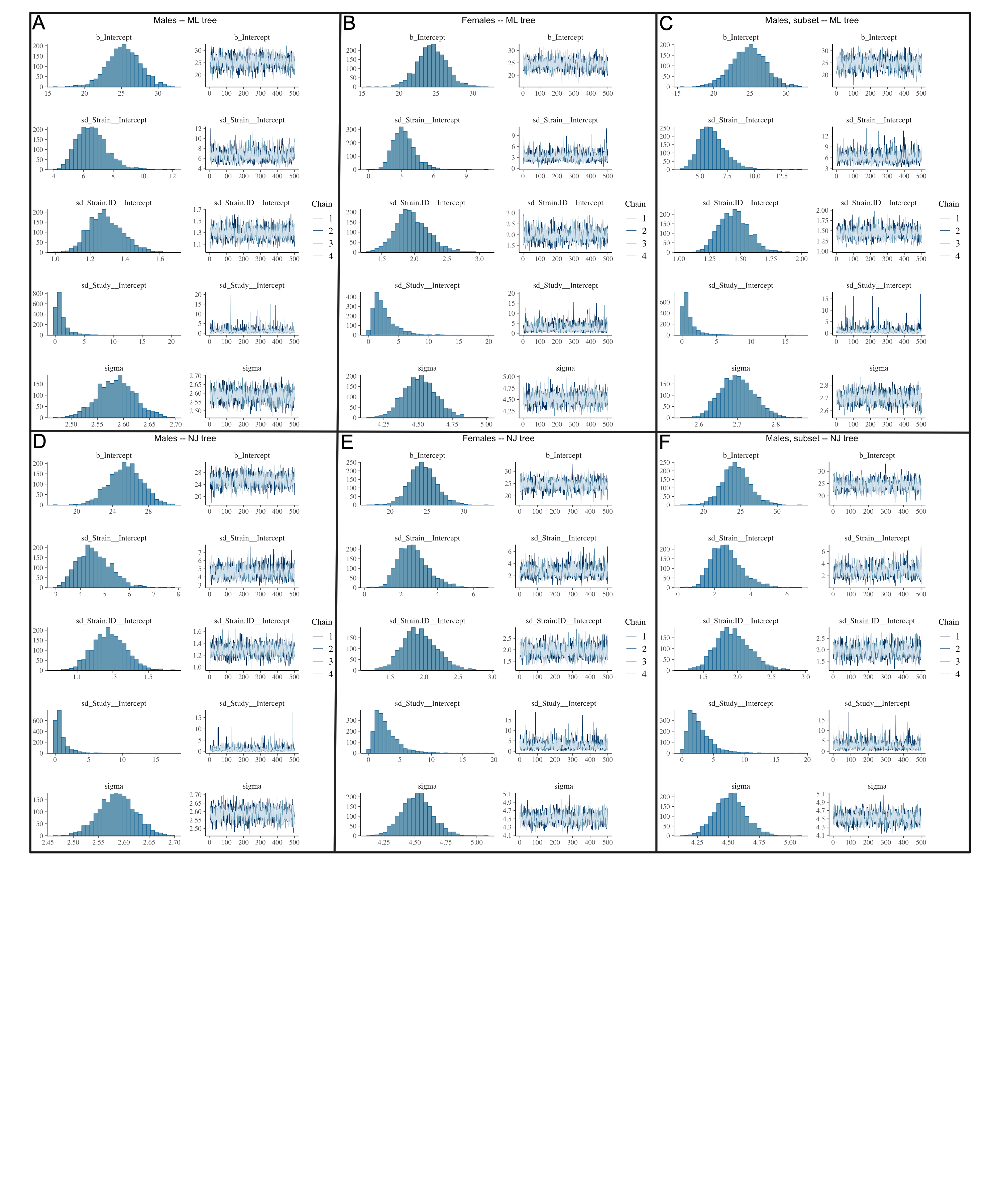

Supplement: iyaf251_Supplementary_Data [file iyaf251_supplementary_data.zip › Figure_S4_GENETICS-2025-308628.png]

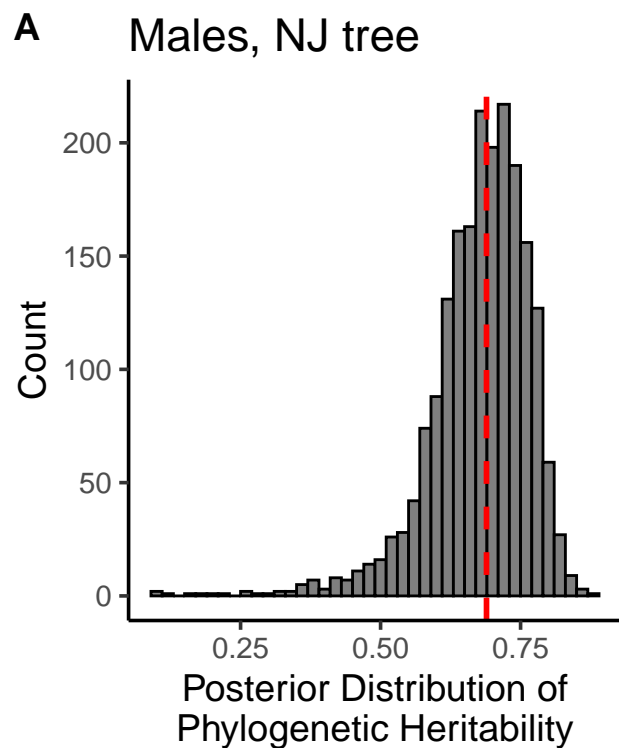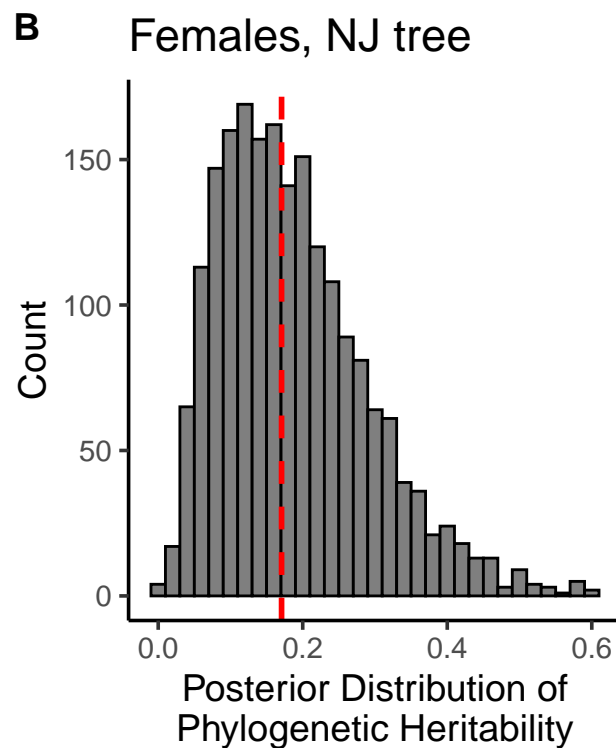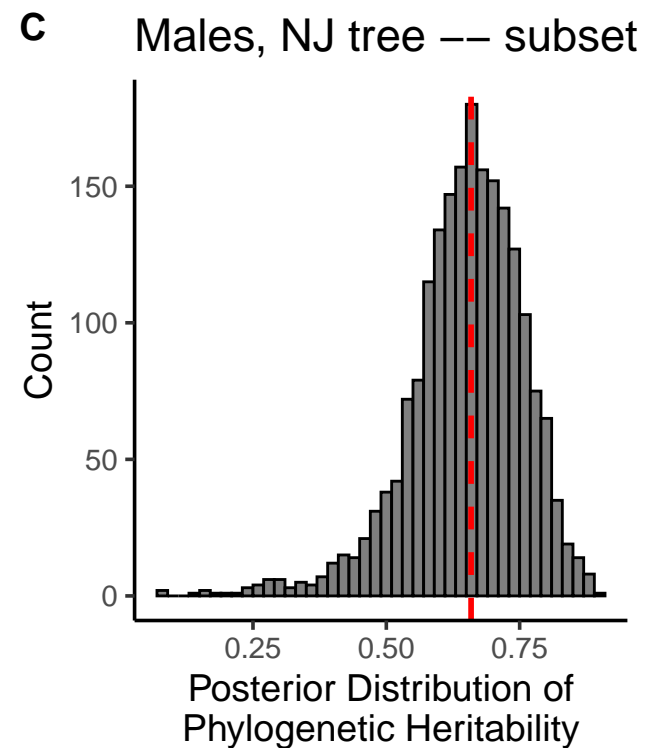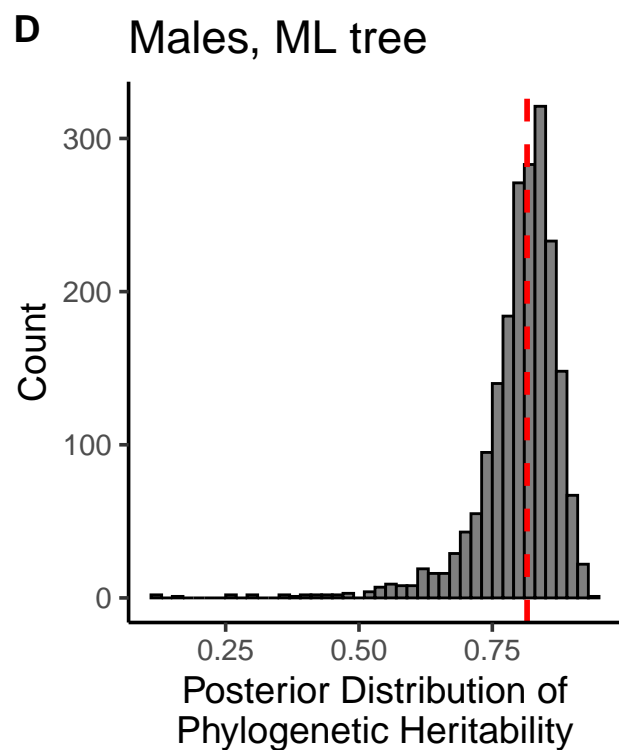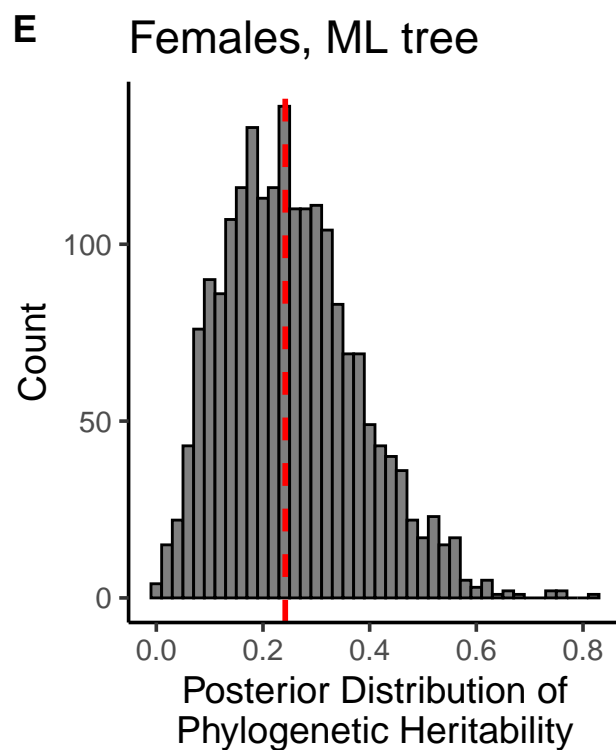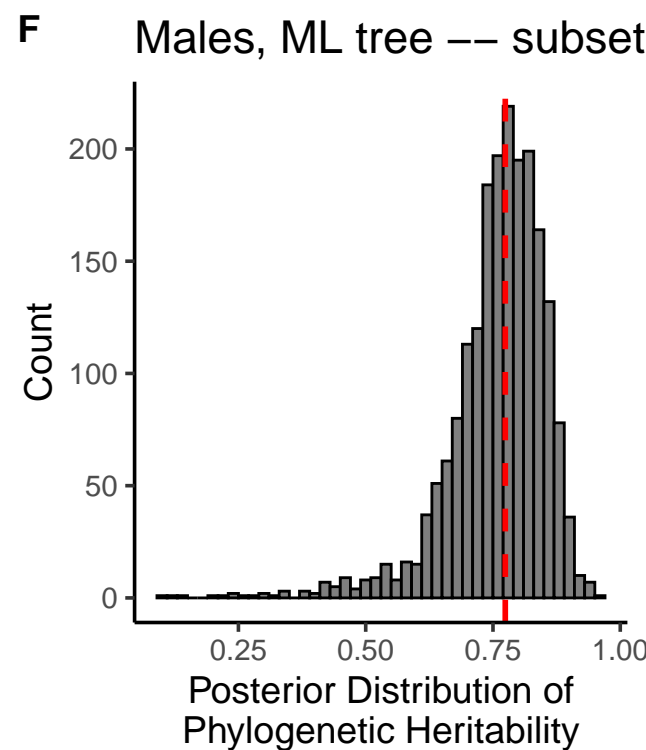

Supplement: iyaf251_Supplementary_Data [file iyaf251_supplementary_data.zip › Figure_S6_GENETICS-2025-308628.pdf]

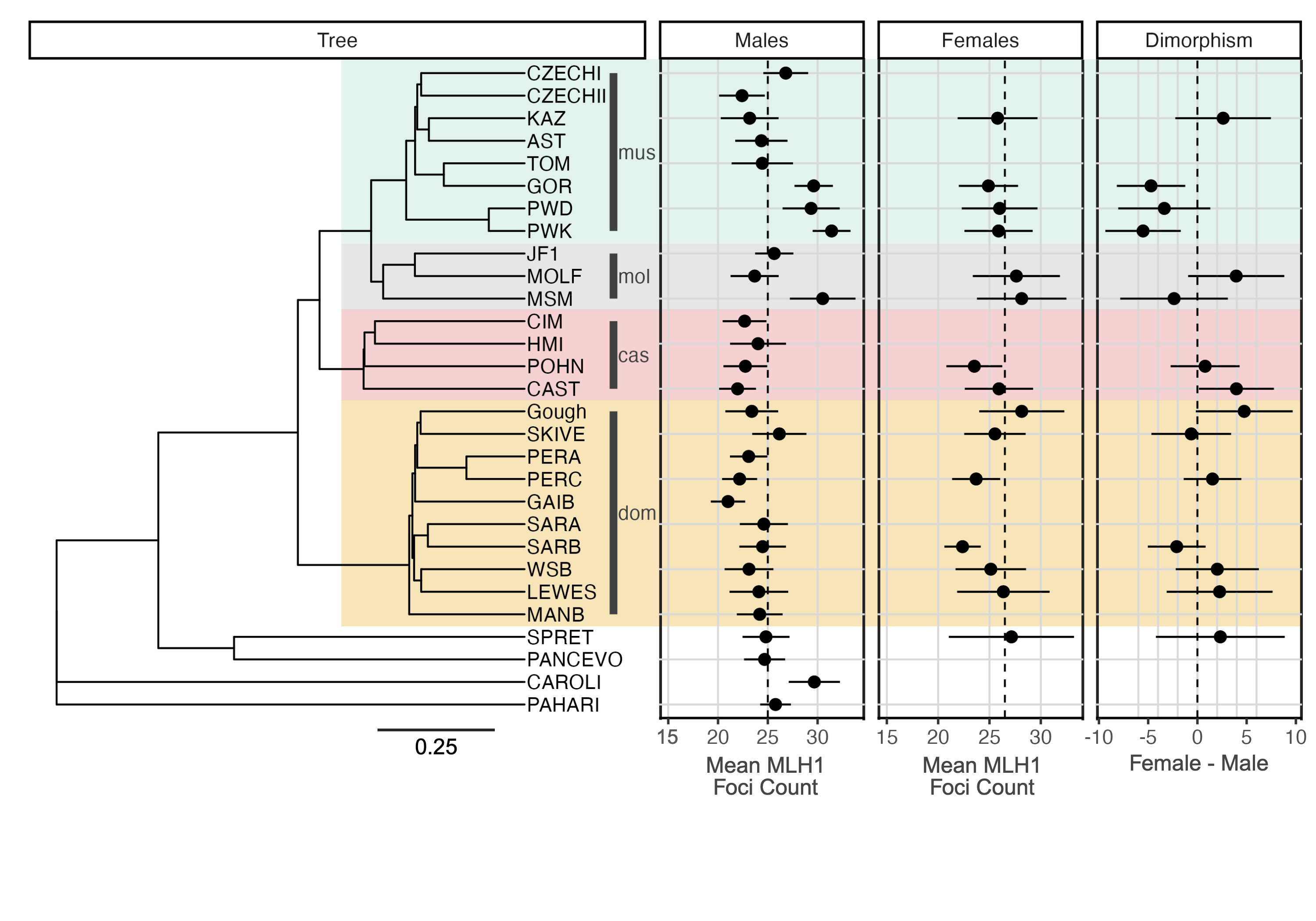

Supplement: iyaf251_Supplementary_Data [file iyaf251_supplementary_data.zip › Figure_S7_GENETICS-2025-308628.png]
